# Glutamate Metabotropic Receptor Type 3 (mGlu3) Localization in the Rat Prelimbic Medial Prefrontal Cortex

**DOI:** 10.3389/fnana.2022.849937

**Published:** 2022-04-04

**Authors:** Elizabeth Woo, Dibyadeep Datta, Amy F. T. Arnsten

**Affiliations:** Department of Neuroscience, Yale University School of Medicine, New Haven, CT, United States

**Keywords:** mGlu3, NAAG, inflammation, schizophrenia, prefrontal cortex

## Abstract

Metabotropic glutamate receptors type 3 (mGlu3, encoded by *GRM3*) are increasingly related to cognitive functioning, including the working memory operations of the prefrontal cortex (PFC). In rhesus monkeys, mGlu3 are most commonly expressed on glia (36%), but are also very prominent on layer III dendritic spines (23%) in the dorsolateral PFC (dlPFC) where they enhance working memory-related neuronal firing. In contrast, mGlu2 are predominately presynaptic in layer III of macaque dlPFC, indicating a pre- vs. post-synaptic dissociation by receptor subtype. The current study examined the cellular and subcellular localizations of mGlu3 in the rat prelimbic medial PFC (PL mPFC), a region needed for spatial working memory performance in rodents. Multiple label immunofluorescence demonstrated mGlu3 expression in neurons and astrocytes, with rare labeling in microglia. Immunoelectron microscopy of layers III and V found that the predominant location for mGlu3 was on axons (layer III: 35.9%; layer V: 44.1%), with labeling especially prominent within the intervaricose segments distant from axon terminals. mGlu3 were also found on glia (likely astrocytes), throughout the glial membrane (layer III: 28.2%; layer V: 29.5%). Importantly, mGlu3 could be seen on dendritic spines, especially in layer III (layer III: 15.6%; layer V: 8.2%), with minor labeling on dendrites. These data show that there are some similarities between mGlu3 expression in rat PL mPFC and macaque dlPFC, but the spine expression enriches and differentiates in the more recently evolved primate dlPFC.

## Introduction

Recent data indicate that metabotropic glutamate receptor type 3 (mGlu3, encoded by *GRM3*) plays an important role in the cognitive function of the prefrontal cortices (PFC). Genetic alterations in *GRM3* are a replicated risk for schizophrenia in genome-wide association studies (Saini et al., 2017), and the reductions in mGlu3 signaling are associated with inefficient activation of the dorsolateral PFC (dlPFC) activity (Egan et al., 2004; Harrison et al., 2008; Jablensky et al., 2011; Chang et al., 2015; Zink et al., 2020), the brain region needed for working memory, cognitive control, and abstract reasoning (Goldman-Rakic, 1995; Courtney et al., 1998; Szczepanski and Knight, 2014). Research from animal models also indicates a strong role for mGlu3s in cognitive function (Jin et al., 2018; Neale and Olszewski, 2019), warranting further understanding of their cellular actions. However, there may be important species differences that must be identified when using animal models to address etiological and therapeutic mechanisms. The current study examined the localization of mGlu3 in rat medial PFC (mPFC), a common rodent model, for comparison to a previous study of the macaque dlPFC.

Metabotropic glutamate receptors type 3 are not only stimulated by glutamate, but also by *N*-acetyl-aspartyl-glutamate (NAAG), which is co-released with glutamate and catabolized by glutamate carboxypeptidase II (GCPII) (Coyle, 1997; Neale, 2011; Vornov et al., 2016). mGlu3s are thought to have neuroprotective actions through expression on both glia and neurons (Spampinato et al., 2018). mGlu3s are expressed on astrocytes across all developmental stages as well as following injury (Schools and Kimelberg, 1999; Mudo et al., 2007). For instance, mGlu3s on astrocytes promote glutamate uptake from the synapse, thus limiting glutamate’s synaptic actions as well as decreasing glutamate spread (Aronica et al., 2003). mGlu3s on microglia have been implicated in mediating neuroprotection by preventing microglial transition into a neurotoxic phenotype, in contrast with the neurotoxic role of mGlu2 (Taylor et al., 2005; Mudo et al., 2007; Berger et al., 2012). mGlu3 expression on neurons is generally considered to be presynaptic, where they reduce glutamate release, complementing their actions on astrocytes and providing negative feedback on glutamate signaling (Sanabria et al., 2004; Zhong et al., 2006). However, immunoelectron microscopic (immunoEM) analyses have shown that mGlu3 also can have a post-synaptic location in the rodent brain, e.g., in the dentate gyrus (Tamaru et al., 2001). Although immunoEM is invaluable for subcellular localization of proteins, the ultrastructural locations of mGlu3 in the rodent PFC are not known.

Rodent models of PFC cognitive disorders have focused on the mPFC, as rodents do not have dlPFC (Wise, 2008), and the mPFC shares some key characteristics with primate dlPFC. For example, lesions to the mPFC, especially of the prelimbic (PL) subregion, impair working memory performance (Larsen and Divac, 1978), and PL neurons show brief persistent firing (∼1 s) during the delay period needed to sustain information in working memory over the delay period in working memory tasks (Yang et al., 2014). Although dlPFC neurons in macaques are able to sustain persistent firing for much longer intervals (e.g., ∼15 s) (Fuster and Alexander, 1971), the qualitative similarities suggest that rodent mPFC may express many of the primordial characteristics that have expanded over cortical evolution. Thus, understanding this mPFC subregion and its similarities and differences with primate dlPFC is important for knowing the strengths and limitations of the rodent model.

Our recent study of the rhesus monkey dlPFC has found that mGlu3 are not only localized on astrocytes, as expected, but also on dendritic spines (Jin et al., 2018) in the layer III microcircuits that generate the persistent firing (Goldman-Rakic, 1995) and gamma bursts (Bastos et al., 2018) underlying working memory. There were surprisingly few mGlu3 at the presynaptic sites, except at locations distal from the synapse near axonal mitochondria (Jin et al., 2018). Instead, the presynaptic receptors in macaque layer III dlPFC were exclusively mGlu2 (Jin et al., 2018). Physiological studies showed that mGlu3 stimulation with NAAG strengthened network connectivity and persistent firing during working memory by inhibiting cAMP opening of the nearby K^+^ channels (Jin et al., 2018). Loss of this capability, e.g., due to genetic insults, would weaken dlPFC firing and may contribute to the cognitive deficits seen in humans with inadequate mGlu3 signaling (Arnsten and Wang, 2020). However, it is not known if there are post-synaptic mGlu3 in rat PL mPFC that may play a similar role or if neuronal mGlu3 in rodent mPFC are exclusively presynaptic. The current study used multiple-label immunofluorescence (IF) coupled with confocal imaging, and immunoEM to localize mGlu3 on neurons, astrocytes, and microglia in rat PL mPFC and to examine their ultrastructural localization at pre- vs. post-synaptic locations on the neurons.

## Materials and Methods

All methods followed NIH and USDA guidelines and were approved by the Yale IACUC.

### Subjects and Tissue Preparation

A total of 5 young adult male (3–4 months) Sprague–Dawley rats (purchased from Envigo; Indianapolis, IN) were used in the present study, out of which 2 rats were used for the multiple-label IF and 3 were used for the quantitative immunoelectron microscopy experiments. Given the intensive nature of the immunoEM, and thus the small numbers of animals used, the work focused on male rats. The age of the rats was comparable to that of the monkeys used in the previous study, i.e., young adults (Jin et al., 2018). Rats were deeply anesthetized with sodium pentobarbital (100 mg/kg, i.v.) and were sacrificed by exsanguination while being perfused transcardially first with 0.1 M phosphate buffer (PB; pH 7.4) for 1 min and then with 4% PFA/0.05% glutaraldehyde plus 15% picric acid in 0.1 M PB (pH 7.4) for 15 min. All perfusates were administered ice-cold. The brains were removed and stored in 0.1 M PB overnight (O/N) at 4°C. The frontal block containing PL mPFC was cut serially in the coronal plane into 70-μm thick sections on a Vibratome (Leica). Sections were collected over the full rostrocaudal extent of the mPFC. All sections used for this study were selected at 2.7–3.2 mm anterior to Bregma (Paxinos and Watson, 1997).

### mGlu3 Antibody

For mGlu3 immunohistochemistry, a well-characterized rabbit polyclonal antibody raised against a synthetic peptide corresponding to the N-terminal (extracellular) domain of human mGlu3 and purified by peptide immunogen affinity column was obtained commercially (G1545, Sigma-Aldrich) for use in this study. The immunizing peptide demonstrates 100% homology with the rat gene and recognizes a sequence that is distinct from mGlu2. This antibody has been extensively tested and shown by others to specifically recognize mGlu3 in the human brain using both immunohistochemical and immunocytochemical approaches. The specificity of this antibody has also been confirmed in *in vitro* assays and by Western blotting (WB) using small interfering RNA for genetic suppression of mGlu3 expression in rodent hypothalamic and cortical cultures (Park et al., 2011; Wang et al., 2012). This antibody has been validated by our group in monkey dlPFC using immunohistochemistry and immunoEM, with complementary expression patterns differing from mGlu2, which would be consistent with its specificity for mGlu3 (Jin et al., 2018). However, it should be noted that the company refused to disclose the immunizing sequence and thus the sequence could not be independently verified. Also the antibody has not been assessed for potential cross-reactivity with mGlu2 in mGlu2 knockout mice, which would substantiate its selectivity.

### Multiple-Label Immunofluorescence and Confocal Imaging

Immunofluorescence staining was carried out on free-floating sections. Antigen retrieval was performed with 2x Antigen Unmasking Solution Citrate Buffer pH 6.0 (Vector Laboratories, H-3300-250) in a steam cooker for 40 min at a high temperature. The free-floating sections were left to cool for 15 min at RT, as stated in the manufacturer’s instructions. After washing the sections in deionized water (2 × 5 min), followed by tap water (1 × 5 min), they were transferred to 1X TBS for 10 min. Sections were blocked for 1 h at RT in 1X TBS containing 5% bovine serum albumin, 2% Triton X-100, and 10% normal goat serum. Sections were incubated for 48 h at 4°C with specific primary antibodies: GFAP (1:500, BioLegend, Cat# 829401) or ionized calcium-binding adaptor molecule 1 (Iba1; 1:500, Synaptic Systems, Cat#234009), MAP2 (1:700, Novus Biologicals, Cat#NBP1-92711), and mGlu3 (see above) in dilution buffer (2% bovine serum albumin, 2% Triton X-100, and 1% normal goat serum). Appropriate secondary antibodies in dilution buffer were used at 1:1,000, overnight at 4°C (goat *anti*-mouse AF488, goat *anti*-chicken, Invitrogen, Cat#A32723; Cat#32932; horse *anti*-rabbit IgG antibody biotinylated, FisherSci, Cat#NC0018276). All subsequent steps were performed in the dark and at RT. The sections were first washed in 1X TBS (pH 7.4, 4 × 15 min), followed by incubation in Streptavidin, AF546 conjugate (0.5 μg/ml, Thermo Fisher Scientific, Cat#S11225) for 30 min. The sections were washed in 1X TBS (pH 7.4, 3 × 5 min) followed by a 4-min incubation in 70% ethanol with 0.3% Sudan Black B (MP Biomedicals, Cat# 4197-25-5) to decrease autofluorescence from lipofuscin. The sections were then washed in 0.02% Tween in 1X TBS (3 × 5 min) followed by washing in 1X TBS (1 × 5 min) and counterstained with Hoechst 33342 for 10 min (1:10,000, Thermo Fisher, Cat# H3570). The sections were washed in 1X TBS (pH 7.4, 3 × 10 min) before mounting onto slides using ProLong Gold Antifade Mountant (Invitrogen, Cat# P36930).

**FIGURE 1:**
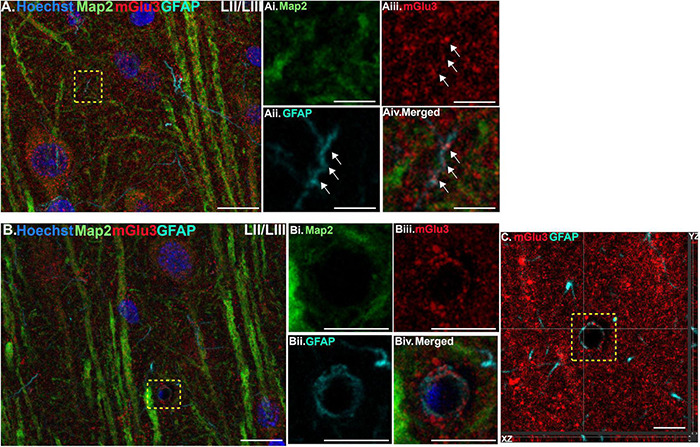
Multiple label immunofluorescence of mGlu3 labeling in astrocytes in layer II/III of rat PL mPFC. Tissue was labeled with antibodies recognizing the astrocytic protein GFAP (cyan), mGlu3 (red), the dendritic marker Map2 (green), as well as the nuclear Hoechst stain (blue). **(A)** A region of layer II/III that is diffusely labeled for mGlu3. Map2 positive dendrites are positive for mGlu3. The yellow box demarcates a GFAP-positive astrocytic process. **(Ai–iv)** Magnified area outlined by the yellow box in A shows a thin astrocytic process (traced by the white arrows) that is mGlu3 and GFAP positive and Map2 negative. **(B)** Expression of mGlu3 (red) is evident in pyramidal-shaped neurons, as seen by co-localization of Map2 and mGlu3, and diffusely across the neuropil. There is a small blood vessel with a GFAP positive astrocytic process overlying the vasculature that contains mGlu3 labeling. **(Bi–iv)** Magnified area of the region delineated by the yellow box in **(B)** showing co-localization of mGlu3 (red) and GFAP (cyan) and the absence of Map2 (green) and Hoechst (blue), consistent with an astrocytic process. **(C)** Orthogonal sectioning of this region (corresponding region of interest from **(B)** outlined by the yellow box). Selected z-stack image shows co-localization of mGlu3 and GFAP (red and cyan) across three different planes for one point, as indicated by the crossed dashed lines. The right-side bar demonstrates labeling in the YZ plane, while the bottom bar represents labeling in the XZ plane. Scale bars: 10 μm **(A,B)**; 2.5 μm **(Ai–iv)**; 5 μm **(Bi–iv)**; 5 μm **(C)**.

**FIGURE 2:**
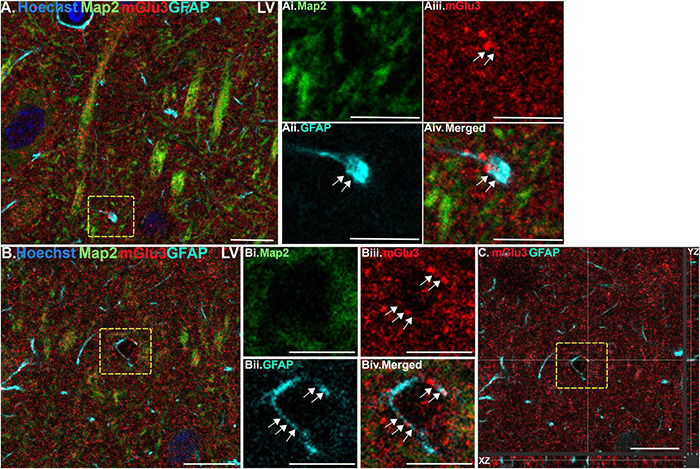
Multiple label immunofluorescence of mGlu3 labeling in astrocytes in layer V of rat PL mPFC. Tissue was labeled with antibodies recognizing the astrocytic protein GFAP (cyan), mGlu3 (red), the dendritic marker Map2 (green), as well as the nuclear Hoechst stain (blue). **(A)** Expression of mGlu3 in the neuropil of Layer V is evident. There is GFAP positive astrocytic process that contains mGlu3 labeling. **(Ai–iv)** Magnified area of the region outlined by the yellow box in **(A)** showing co-localization of mGlu3 (red) and GFAP (cyan) within a bulbous end of a process, without the presence of Map2 (green). **(B)** This is an example of a GFAP positive astrocytic process overlying likely a blood vessel. **(Bi–iv)** Magnified area of the yellow box in **(B)** showing co-localization of mGlu3 and GFAP in a Map2 negative region. **(C)** Orthogonal sectioning of this region (corresponding region of interest from **(B)** outlined by the yellow box). Selected z-stack image shows co-localization of mGlu3 and GFAP (red and cyan) across three different planes for one point, as indicated by the crossed dashed lines. The right-side bar demonstrates labeling in the YZ plane, while the bottom bar represents labeling in the XZ plane. Scale bars: 10 μm **(A,B)**; 5 μm **(Ai–iv,Bi–iv)**; 10 μm **(C)**.

Confocal images were acquired using a Leica TCS SP8 Gated STED 3X super-resolution microscope with HC PL APO 100X/1.40 oil white objective (Leica) and HCX PL APO CS 63X/1.40 oil white objective (Leica). *Z*-stacks were obtained with 0.3-μm steps under laser excitation at 407, 488, 543, and 633 nm. Emission filter bandwidths and sequential scanning acquisition were set up to avoid possible spectral overlap between fluorophores. The prelimbic region for each section was identified by referencing Paxinos and Watson’s *The Rat Brain in Stereotaxic Coordinates*. The confocal *Z*-stacks were processed into maximum intensity *Z*-projections using Fiji and background subtraction, and a rolling ball with a radius of 50 pixels and 100 pixels was applied to all 63X and 100X channels, respectively (applied to the entire panel). Images were labeled and assembled into a figure using Adobe Photoshop CS5 Extended (version 12.0.4 × 64, Adobe Systems Incorporated). To confirm double IF co-localization, the acquired *z*-stacks for all antibody combinations were examined using the orthogonal sectioning function on the Leica LASX software. For one point of interest, three planes of view can be examined (XY, YZ, and XZ planes). Representative orthogonal sectioning views were selected to demonstrate GFAP and mGlu3 co-localization in LIII and LV (Figures 1, 2).

### Immunoelectron Microscopy

#### Pre-embedding Immunocytochemical Labeling

Pre-embedding peroxidase immunolabeling was performed using methods similar to those described in our previous reports (Jin et al., 2018). Following O/N storage in 0.1 M PB at 4°C, free-floating sections were rinsed thoroughly with 0.1 M phosphate buffer saline (PBS; pH 7.4), followed by 50 mM TBS (pH 7.6). They were then treated with 0.5% sodium borohydride in 50 mM TBS for 15 min to quench non-reactive aldehydes. To suppress the non-specific binding of antisera and enhance the penetration of immunoreagants, sections were incubated in 10% normal goat serum and 2% IgG-free bovine serum albumin (BSA) supplemented with 0.1% Triton X-100 detergent in TBS for 2 h at RT. After protein blocking, the sections were incubated with an antibody raised against human mGlu3 (1:100 dilution; 10 μg/ml; Sigma-Aldrich) in TBS for 48 h at 4°C. Next, they were transferred to biotinylated F(ab’)2 fragment goat anti-rabbit IgG (1:500; Jackson ImmunoResearch Laboratories) in TBS for 2 h at RT and then washed in TBS. For immunoperoxidase detection, the sections were incubated with avidin-biotin-peroxidase complex (Vectastain Elite Kit; Vector) for 1 h, washed with TBS followed by tris buffer (TB), and then placed in 0.05% DAB in TB with the addition of 0.01% hydrogen peroxide for 8 min. After several rinses, first with TBS and then with TB followed by PB, the sections were fixed in 1% osmium tetroxide in 0.1 M PB for 30 min. They were then washed thoroughly with 0.1 M PB and counterstained with filtered 1% uranyl acetate prepared in deionized water. Following dehydration with a graded series of ethanol and propylene oxide, the sections were then incubated O/N in a 1:1 mixture of propylene oxide and Durcapan epoxy resin (Fluka). Finally, they were transferred to 100% fresh epoxy resin for 2 h and flat-embedded between a glass microscope slide and coverslip coated with Liquid Release Agent for subsequent polymerization for 48 h at 60°C. Layers I–III and V–VI of prelimbic mPFC were sampled for 1–2 plastic blocks per brain and resectioned into ultrathin (50-nm thick) sections on a Reichert-Jung ultramicrotome using a Diatome diamond knife. Ultrathin sections were collected on individual formvar-supported slot grids and air-dried for subsequent imaging and analysis. Unless otherwise indicated, all chemicals and supplies for electron microscopy experiments were obtained from Sigma-Aldrich and Electron Microscopy Sciences, respectively.

#### Methodological Controls

Omission of the primary antibody and substitution with non-immune rabbit serum abolished all reactivity. Peroxidase label was not detected when bridging secondary antibodies were excluded. Blocking of biotinylated probes with avidin/biotin also abolished the signal. Finally, additional electron microscopic immunocytochemistry performed in monkey dlPFC by our group showed that the antibody used in the present study exhibited a labeling pattern that was a subset of another commercially available and well-characterized antibody (Ab6438; Abcam) raised against the shared C-terminus of mGlu2 and mGlu3 (Jin et al., 2017), consistent with the labeling of mGlu3.

#### Quantitative Analysis of mGlu3 Immunolabeling

Subcellular distributions of mGlu3 peroxidase immunolabeling were determined using approaches similar to those employed extensively by our group (e.g., Jin et al., 2018). Briefly, to exclude penetration artifacts, all analyses were performed on ultrathin sections collected near the surface of the tissue at the interface with the embedding material (e.g., from the 4th to the 10th surface-most section of each block). Peroxidase-labeled material was examined at 80 kV under a Jeol 1010 transmission electron microscope equipped with an AMTAdvantage CCD camera (Advanced Microscopy Techniques). Using a systematic-random approach, 7.4 μm^2^ fields were digitally captured at × 50,000 original magnification (Gatan) from approximately 300–350 μm and 600–700 μm deep to the basal laminar border of layer I for visualization of layer II/III and V, respectively (Gabbott et al., 1997). Approximately 100 micrographs containing a total of ∼400 labeled profiles were obtained for each rat included in the final quantitative dataset (*n* = 3), with a total of 635 micrographs from layer II/III and 560 from layer V. Profiles containing mGlu3 reactivity were classified as either neuronal (dendrites, spines, axons, and axon terminals) or glial based on criteria summarized in Peters et al. (1991). Immunoperoxidase labeling was considered positive when the electron-dense reaction product in individual profiles was greater than that observed in morphologically similar profiles in the neuropil. Each electron micrograph was adjusted for brightness, contrast, and sharpness (applied to the entire panel), and pseudocolored for clarity using Adobe Photoshop CS5 Extended (version 12.0.4 × 64, Adobe Systems Incorporated).

### Statistical Analyses

Statistical analyses were performed using IBM SPSS Statistics software, version 20 (IBM Corporation). All data followed a normal distribution (one-sample Kolmogorov–Smirnov test, *p* > 0.05), and parametric tests were applied accordingly. A paired *t*-test compared spine labeling in layer II/III vs. V. Significance was defined as *p* ≤ 0.05 for all statistical tests. All data are reported as the mean ± SEM.

## Results

### Multiple-Label Immunofluorescence

**FIGURE 3:**
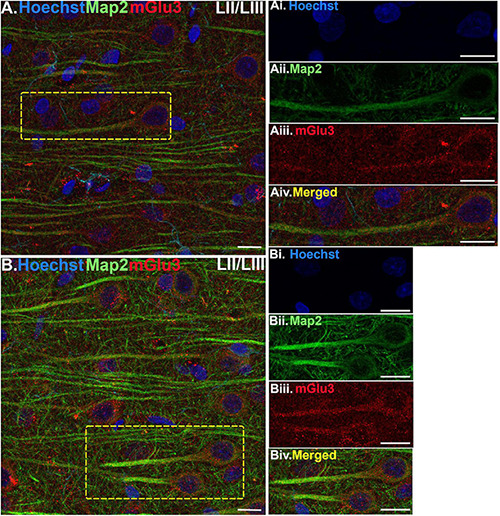
Multiple label immunofluorescence of mGlu3 labeling in neurons in layer II/III of rat PL mPFC. Tissue was labeled with antibodies recognizing mGlu3 (red), the dendritic marker Map2 (green), as well as the nuclear Hoechst stain (blue). **(A,B)** In layer II/III, mGlu3 expression (red) is seen diffusely across the neuropil, with labeling within the perisomatic regions of putative pyramidal neurons as well as the apical dendrites. One representative neuron is outlined by the yellow dashed box in **(A)**, and two representative neurons are outlined in **(B)**. **(Ai–iv,Bi–iv)** Correspond to the boxed areas in **(A,B)**. In both **(Aii,iii,Bii,iii)** mGlu3 labeling clearly outlines the apical dendrite, as demarcated by Map2 (green) and surrounds the nucleus (blue). Scale bars: 10 μm.

**FIGURE 4:**
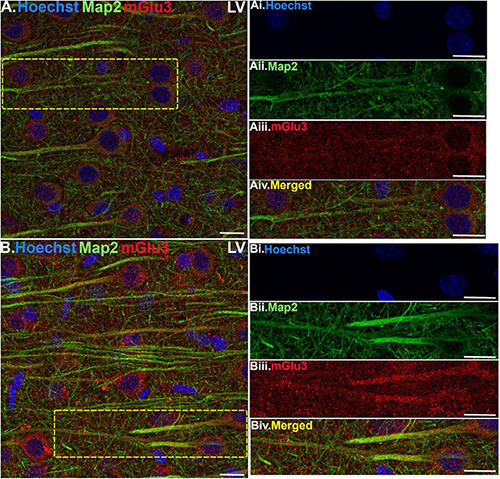
Multiple label immunofluorescence of mGlu3 labeling in neurons in layer V of rat PL mPFC. Tissue was labeled with antibodies recognizing mGlu3 (red), the dendritic marker Map2 (green), as well as the nuclear Hoechst stain (blue). **(A,B)** In layer V, mGlu3 expression (red) is seen diffusely across the neuropil, with labeling within the perisomatic regions of putative pyramidal neurons as well as the apical dendrites. Two representative neurons are outlined by the yellow dashed boxes in **(A,B)**. **(Ai–iv,Bi–iv)** Correspond to the boxed areas in **(A,B)**. In both **(Aii,iii,Bii,iii)** mGlu3 labeling clearly outlines the apical dendrites, as demarcated by Map2 (green) and surrounds the nucleus (blue). Scale bars: 10 μm.

Multiple-label IF was used to determine mGlu3 expression across different cell types in the rat PL mPFC. To localize mGlu3 expression on neurons, we used microtubule-associated protein 2 to label both the perikarya and neuronal dendrites. Dense mGlu3 neuropil labeling can be appreciated in layer II/III (Figure 3) and layer V (Figure 4) of young adult rat PL mPFC. Specifically, mGlu3 labeling is evident within the perikaryon as well as along the apical dendrites of putative pyramidal cells, as demonstrated by examples of mGlu3 and Map2 co-localization in layer II/III (Figure 3) and layer V (Figure 4). For example, Figure 3B demonstrates an area within layer II/III showing several putative pyramidal cells with punctate-like mGlu3 labeling along the apical dendrite and within the perikaryon.

We assessed mGlu3 expression in astrocytes by staining for glial fibrillary acidic protein (GFAP). There was a diffuse expression of mGlu3 on the processes of astrocytes, as opposed to the astrocytic soma, in both layer II/III (Figure 1) and layer V (Figure 2). For example, in Figure 1B, there is mGlu3 labeling on an astrocytic process likely enveloping a blood vessel in layer II/III, which is almost entirely covered by astrocytic endfeet (Mathiisen et al., 2010). A similar relationship is seen in layer V in Figure 2B, where a GFAP positive astrocytic process containing mGlu3 labeling is overlying vasculature. Figure 1A captures an example of mGlu3 labeling in a thin astrocytic process traversing through layer II/III of the neuropil.

**FIGURE 5:**
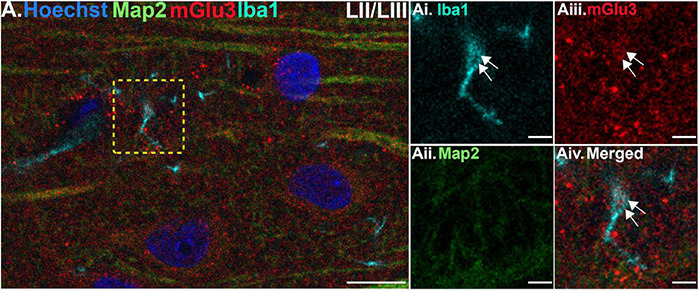
Multiple label immunofluorescence of mGlu3 labeling in microglia in layer II/III of rat PL mPFC. Tissue was labeled with antibodies recognizing the microglial protein Iba1 (cyan), mGlu3 (red), the dendritic marker Map2 (green), as well as the nuclear Hoechst stain (blue). **(A)** There are diffuse Iba1 positive microglial processes across the neuropil in layer III. **(Ai–iv)** Magnified area of the yellow box in **(A)**, showing an Iba1 positive microglial process (cyan) co-localized with mGlu3 (red), indicated by white arrows. Scale bars: 10 μm **(A)**, 2.5 μm **(Ai–iv)**.

**FIGURE 6:**
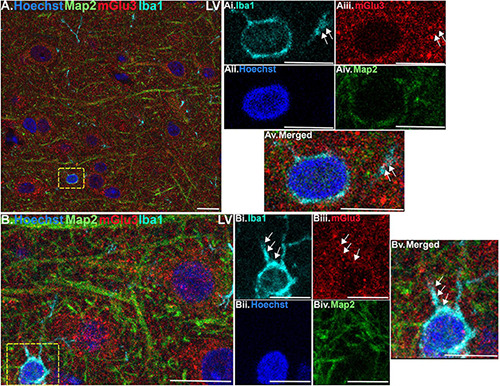
Multiple label immunofluorescence of mGlu3 labeling in microglia in layer V of rat PL mPFC. Tissue was labeled with antibodies recognizing the microglial protein Iba1 (cyan), mGlu3 (red), the dendritic marker Map2 (green), as well as the nuclear Hoechst stain (blue). **(A)** Similarly, to layer II/III, there are diffuse Iba1 positive microglial processes throughout the neuropil in layer V. **(Ai–v)** Magnified area of the yellow box in **(A)**, showing an Iba1 positive microglial soma surrounding the Hoechst positive nucleus (blue) and emanating processes (cyan). The process contains mGlu3 (red) indicated by white arrows, which are Map2 negative. **(B)** In another region of layer V, there is an Iba1 positive microglial soma (cyan) with two visible processes in this plane. **(Bi–v)** Magnified area of the yellow box in **(B)**, demonstrates mGlu3 localization within the processes extending from the soma, as demarcated by the white arrows. Scale bars, 10 μm **(A,B)**; 5 μm **(Ai–v,Bi–v)**.

Finally, we stained the tissue with Iba1, a marker for microglia and macrophages, to determine mGlu3 expression on microglia. Microglia were relatively rare in this young, healthy tissue, but there were examples of mGlu3 labeling in microglial processes in layer II/III (Figure 5) and layer V (Figure 6).

The finding that mGlu3 were predominately expressed on astrocytic and microglial processes rather than in their cell bodies is a challenge, given the relatively low resolution of multiple-label IF, and the small size of these glial processes. Observing mGlu3 labeling along the length of a glial process requires its fortuitous capture by a coincident plane of section. Given the relatively small numbers of microglia expressed in our tissue, and the limits on capturing labeling in fine glial processes, quantitative comparisons of astrocytic vs. microglial mGlu3 labeling were not performed.

### Immunoelectron Microscopy

**FIGURE 7:**
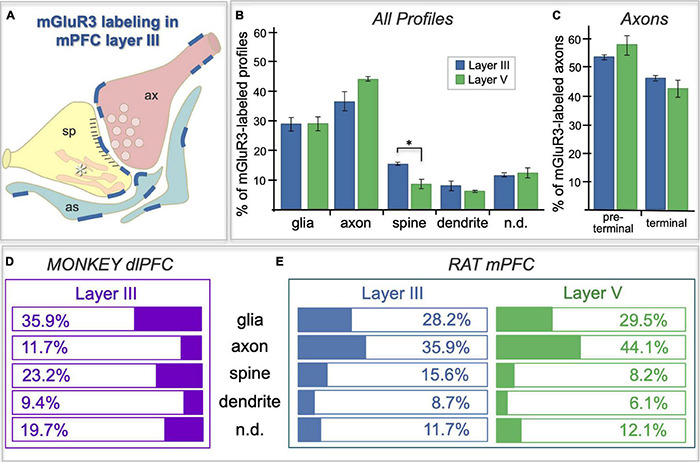
Frequency of mGlu3-labeled profiles in layer II/III vs. V of rat PL mPFC, and a comparison to the pattern in macaque. **(A)** Schematic of the localization patterns of mGlu3 in rat PL mPFC layer II/III; labeling represented by dark blue lines. Sp = spine, ax = axon terminal, as = astrocyte, asterisk indicates the calcium-containing SER spine apparatus. **(B)** Bar graphs of the average frequency of mGlu3-containing cellular profile types, expressed as a percentage of the total number of immunoreactive profiles, in layer II/III vs. V of rat PFC. In addition to the classical astroglial expression, mGlu3 are concentrated presynaptically in axons in both layers. mGlu3 are also expressed in spines, with significantly more expression in layer II/III than in layer V. **(C)** Immunoreactive axonal profiles in rat PFC are further classified into preterminal and terminal categories. A numerically larger proportion of mGlu3-labeled axons are preterminal compared to terminal in the two layers, which did not differ from one another. For **(B,C)**, group results are expressed as the mean ± SEM. *p < 0.05. *n* = 3 rats. **(D,E)** Comparison of mGlu3 expression patterns in rhesus macaque layer III dlPFC **(D)** with that in rat PL mPFC layers II/III and V **(E)**. mGlu3 predominate on glia (presumed astrocytes) and spines in monkey, but on axons in rat.

ImmunoEM was used to analyze the subcellular localization of mGlu3 in layer II/III and layer V of rat PL mPFC. The results are summarized in Figure 7 and Table 1, showing that mGlu3 labeling was predominately presynaptic on axons and axon terminals, with substantial labeling of glia and some expression in dendritic spines in layer II/III, but very modest levels of dendritic spine expression in layer V. It should be noted that the apical dendrites of layer V traverse through layer II/III, and thus some of the labeling in this layer may include contributions from these deeper cells in addition to those residing in layer II/III.

**TABLE 1:** Quantitative assessment of mGlu3 immunoreactivity in rat mPFC PL layer II/III and layer V neuropil.

	Axon *n*%	Spine *n*%	Dendrite *n*%	Glia *n*%	n.d. *n*%
**Rat 1**					
Layer II/III	46 35.1%	19 14.5%	8 6.1%	43 32.8%	15 11.5%
Layer V	66 44.6%	15 10.1%	9 6.1%	36 24.3%	22 14.9%
**Rat 2**					
Layer II/III	90 42.9%	31 14.8%	14 6.7%	53 25.2%	22 10.5%
Layer V	79 42.7%	19 10.3%	12 6.5%	58 31.3%	17 9.2%
**Rat 3**					
Layer II/III	92 31.3%	49 16.7%	33 11.2%	83 28.2%	37 12.6%
Layer V	102 44.9%	12 5.3%	13 5.7%	71 31.3%	29 12.8%
**Total**					
Layer II/III	228 35.9%	99 15.6%	55 8.7%	179 28.2%	74 11.7%
Layer V	247 44.1%	46 8.2%	34 6.1%	165 29.5%	68 12.1%

*Prevalence of mGlu3 in cellular compartments.*

**FIGURE 8:**
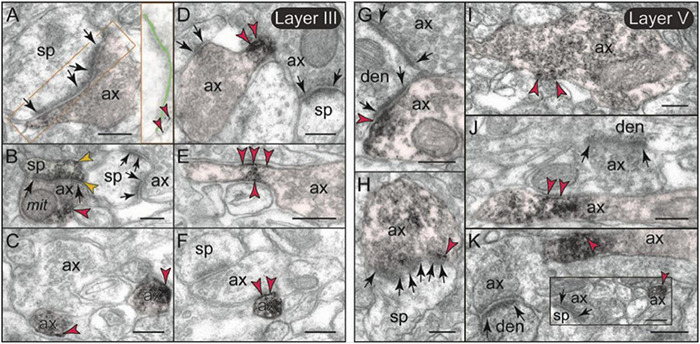
Presynaptic expression of mGlu3 in layers II/III and V of rat PFC. **(A,B)** In layer II/III, a moderate presynaptic component of mGlu3 is captured in glutamatergic-like axons terminals establishing asymmetric synapses both near the synaptic active zone **(A)**, and extrasynaptically near a mitochondrion **(B)**. One of the two spines in **(B)** is also labeled. The synaptic cleft is magnified and highlighted with light green shading in **(A)** to better show the location of mGlu3 labeling in relationship to the synapse. **(C–F)** Bundled preterminal axons typically express mGlu3 at the plasma membrane. **(G–K)** In layer V, presynaptic mGlu3 can be visualized perisynaptically on membranes flanking the synapse **(G,H)**, and near presynaptic vesicles **(G–I)**, with an additional component in preterminal axons **(J,K)**. Preterminal axons are shown both in cross-section (e.g., **C**), and in longitudinal section (e.g., **D**) for comparison. Synapses are between arrows. Red arrowheads point to mGlu3 immunoreactivity on axons; yellow arrowheads in **(B)** indicate additional mGlu3 labeling on a spine. Profiles are pseudocolored for clarity. as, astrocyte; ax, axon; den, dendrite; mit, mitochondrion; sp, spine. Scale bar: 200 nm.

The labeling of mGlu3 in rat PL mPFC was most prominent in axons (Figures 7B,E), and examples of axonal labeling can be seen in Figure 8. Axonal labeling could be seen near presynaptic release sites in layer II/III (Figure 8A) and V (Figures 8G,H), but was most prominent in axon segments prior to the terminal region (Figures 7C, 8B), especially within intervaricose segments (Figures 8C–F,I–K). mGlu3 labeling at the intervaricose segments may regulate glutamate volume transmission from non-synaptic sites.

**FIGURE 9:**
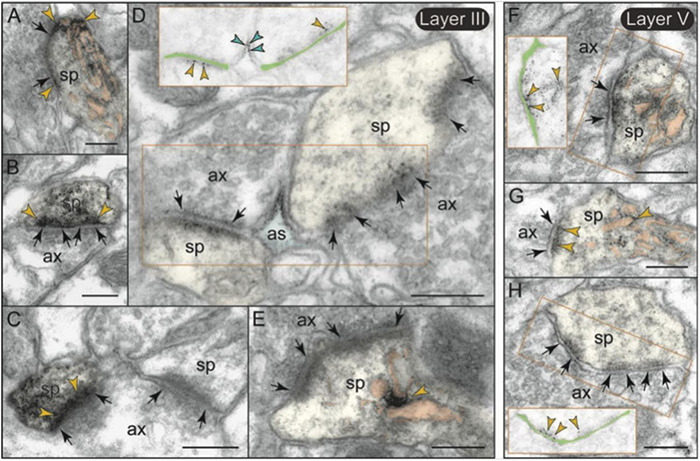
Post-synaptic expression of mGlu3 in spines in layersII/III and V of rat PFC. **(A–E)** In layer II/III, mGlu3 are found post-synaptic to glutamatergic-like synapses where it appears perisynaptically **(A,B)**, but is also often captured next to (or over) the spine apparatus (pseudocolored salmon pink; **A,E**), and within the synapse *per se* **(B–D)**. A non-labeled axospinous synapse is shown in **(C)** for comparison. Note in **(D)** the non-uniform expression of mGlu3 along the length of the perforated synapse. An astrocytic leaflet is also labeled in **(D)**. **(F–H)** mGlu3-reactive dendritic spines in layer V also exhibit prevalent labeling in association with the spine apparatus, and within the synapse *per se*. The insets in **(D,F,H)** are edited to facilitate visualization of immunoperoxidase reaction product at the synapse. Light green shading shows the location of the synaptic cleft. Synapses are between arrows. Yellow arrowheads point to mGlu3 immunoreactivity in spines. Profiles are pseudocolored for clarity. as, astrocyte; ax, axon; den, dendrite; sp, spine. Scale bars: 200 nm.

mGlu3 labeling was also observed at post-synaptic locations on spines, particularly in layer II/III (Figure 7E), although spine labeling was much less than that in axons (Figures 7B,E). Examples of mGlu3 spine labeling are shown in Figure 9, including labeling within and near the post-synaptic density (PSD; Figures 9A–D,F–H), as well as near the calcium-containing smooth endoplasmic reticulum (SER) spine apparatus (Figures 9A,E–G), where it may regulate cAMP drive on internal calcium release. For example, Figure 9A shows mGlu3 on the plasma membrane of a layer II/III spine near both the synapse and the spine apparatus (pseudocolored in pink). There was significantly greater spine labeling in layer II/III than in layer V by almost two-fold (Figure 7E; *p* = 0.039, paired *t*-test).

**FIGURE 10:**
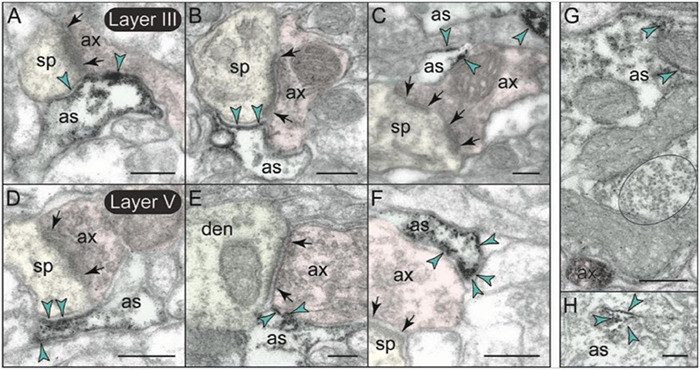
Astrocytic expression of mGlu3 in layers II/III and V of rat PFC. **(A–F)** In both layers II/III and V of rat PFC, astrocytic mGlu3 are primarily localized on the plasma membrane of astrocytic leaflets enwrapping asymmetric synapses, where it is especially concentrated near the synapse. **(G,H)** mGlu3 are also found at sites remote from the synapse, including extrasynaptically on the plasma membrane of astrocytic processes **(G)**, and very delicate labeling in the soma in association with the synthetic machinery, e.g., granular endoplasmic reticulum **(H)**. Note in **(G)** the prominent bundle of astrocytic intermediate filaments (black oval); an intervaricose axon is also labeled at the bottom of the image. Synapses are between arrows. Blue arrowheads point to mGlu3 immunoreactivity on glia. Profiles are pseudocolored for clarity. as, likely astrocyte; ax, axon; den, dendrite; sp, spine. Scale bars: 200 nm.

Metabotropic glutamate receptors type 3 labeling was also evident in its expected location on glial membranes in both layers II/III and V (Figures 7, 10). mGlu3 labeling could be seen on glial membranes at locations near the synapse (Figures 10A–E), as well as on glial membranes, apparently distant from the synapse (Figures 10F,G). Delicate mGlu3 labeling could also be seen within the glial cell body in association with the synthetic machinery, e.g., the granular endoplasmic reticulum (Figure 10H). Quantitative analyses revealed comparable levels of mGlu3 in glial profiles in both layer II/III and layer V in rat mPFC (Figure 7E).

**FIGURE 11:**
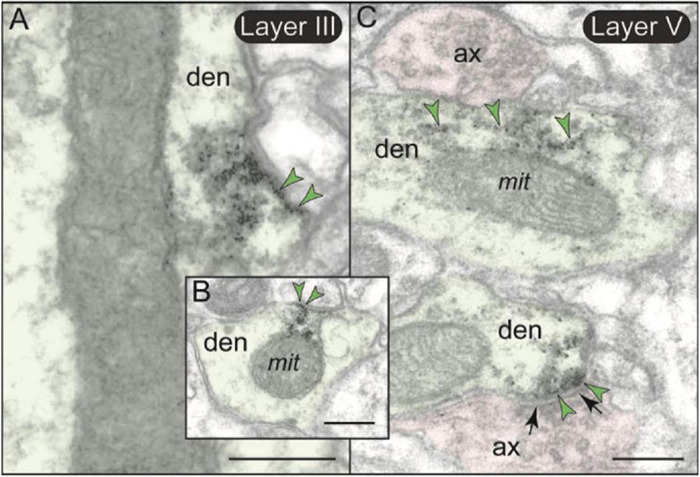
Post-synaptic expression of mGlu3 in dendritic shafts in layers II/III and V of rat PL mPFC. **(A,B)** In both layers II/III and V (but shown here in layer II/III), mGlu3s are visualized on the plasma membranes of dendritic shafts, often on the patch of membrane facing a mitochondrion. It is possible that mGlu3s are regulating cAMP influences on mitochondria at this location, e.g., regulating cAMP drive on internal calcium release near these organelles. However, there are no apparent synapses evident near the mGlu3 labeling. **(C)** mGlu3 labeling can also be seen on microtubules in dendritic shafts in both layers (but shown here in layer V), where it may be trafficking within the dendrite. There is also a limited post-synaptic mGlu3 component associated with glutamatergic-like axodendritic synapses (bottom of image). Synapses are between black arrows. Green arrowheads point to mGlu3 immunoreactivity. Profiles are pseudocolored for clarity. ax, axon; den, dendrite; mit, mitochondrion. Scale bars: 200 nm.

There was also minor mGlu3 post-synaptic labeling on dendritic shafts (Figure 7E). Dendritic mGlu3 labeling was often associated with mitochondria in both layers II/III and V where it may regulate cAMP influences on mitochondrial function (Figures 11A–C). There were also rare instances where dendritic mGlu3 label was associated with a glutamatergic-like synapse (Figure 11C, bottom).

In summary, a comparison of the frequency of mGlu3 expression in primate layer II/III dlPFC vs. rodent PL mPFC is shown in Figures 7D,E. Our previous study (Jin et al., 2018) of layer III dlPFC in the rhesus macaque showed that mGlu3 predominate in astroglial and post-synaptic locations (Figure 7D), with much less labeling in axons (Figure 7D), where mGlu3 expression was limited to axonal mitochondria distant from presynaptic release sites. In contrast, the current study shows that axons are the predominant locations for mGlu3 in rat PL mPFC, although with significant glial and dendritic spine contributions, especially spine labeling in layer II/III. Thus, there are notable differences in mGlu3 localization between macaque layer III dlPFC and rat PL mPFC, but with important similarities that may bridge across species and the PFC subregion.

## Discussion

The current study used multiple-label IF and immunoEM to localize mGlu3 protein in the rat PL mPFC. The data showed that mGlu3 are clearly localized on the neurons and astrocytes, with minor labeling of microglia in young and healthy tissue. Within neurons, the immunoEM showed that mGlu3s are most frequently concentrated in their traditional presynaptic locations in the rat PL mPFC. However, some mGlu3 could also be seen on dendritic spines in PL mPFC, especially in layer II/III, the predominant neuronal site for mGlu3 in layer III primate dlPFC. The localization of mGlu3 on layer III dlPFC dendritic spines is of particular interest, as these receptors strengthen the synaptic connectivity and neuronal firing needed for working memory by inhibiting cAMP-PKA opening of K^+^ channels on spines (Jin et al., 2018). Thus, an enrichment in post-synaptic mGlu3 on spines may arise with the evolutionary expansion of layer III in primate dlPFC, consistent with the important role of these receptors in human cognition.

### Species Differences in mGlu3 Localization

Most reviews of mGlu3 signaling emphasize their expression on presynaptic glutamate terminals and on astrocytes, where they are thought to reduce glutamate signaling by inhibiting release and increasing glutamate uptake, respectively (Hascup et al., 2010). In the current study, these were indeed the predominant locations for mGlu3 in rat PL mPFC, particularly on axons. Axonal labeling included not only presynaptic terminals, where they likely inhibit glutamate release into the synapse, but also in preterminal axonal segments that may regulate glutamate volume release. These data differ greatly from those in primate dlPFC, where axonal type II mGlu receptors are predominately mGlu2, with very little mGlu3 at these presynaptic locations (Jin et al., 2018). Thus in primates, the dissociation of presynaptic mGlu2 vs. post-synaptic mGlu3 may facilitate the development of more effective, selective therapeutics, whilst the location of mGlu3 at both pre- and post-synaptic locations in rodent mPFC may obscure their respective roles through countermanding actions.

There were also species differences in the qualitative pattern of astrocytic labeling, where mGlu3 in rats diffusely label astrocytic membranes, while in monkey dlPFC, mGlu2 display diffuse expression and mGlu3 are concentrated on astrocytic membranes next to the synapse (Jin et al., 2018). Thus, some of the roles of mGlu3 in limiting glutamate signaling in the rodent mPFC appear to be mediated by mGlu2 in the primate dlPFC.

Metabotropic glutamate receptors type 3 expression on spines may be particularly important for higher cognition and cognitive disorders, and thus species differences in this subset of receptors may be particularly important. Studies of the macaque layer III dlPFC show enrichment of mGlu3 on dendritic spines, and mGlu3 stimulation greatly increases the persistent neuronal firing needed for working memory (Jin et al., 2018). mGlu3 message is also enriched in human dlPFC (Carlyle et al., 2017), although the ultrastructural localization of mGlu3 in humans is not known. Given the increasing links between cognitive dysfunction and insults to mGlu3 signaling in humans (Ghose et al., 2009; Zink et al., 2020), it is important to determine whether post-synaptic mGlu3 also exists in rodent mPFC. The current results showed that there are indeed mGlu3 on the dendritic spines in rat mPFC in the exact same subcellular locations as in primate, namely, within the PSD, peri-synaptically, and near the calcium-storing spine apparatus. However, these occurred at a lower frequency than in primates. Thus, spine labeling makes up 23.2% of profiles in layer III of macaque dlPFC, but 15.6% in layer II/III of rat PL mPFC, and only 8.2% in layer V of rat PL mPFC. It is not known which connections are associated with mGlu3-labeled spines, e.g., if they reside on the subset of spines that receive inputs from the association cortices, hippocampus, and/or MD thalamus that are concentrated in layer III (Collins et al., 2018; Canetta et al., 2020), they may strengthen the connections most needed for working memory and attention regulation (Spellman et al., 2015; Bolkan et al., 2017; Schmitt et al., 2017; Halassa and Sherman, 2019). This would be a fascinating area for future research, identifying the projections of mGlu3-expressing pyramidal cells in rodent mPFC to better understand how these neurons may be contributing to circuits that regulate behavior.

Importantly, as mGlu3 generally have opposite effects at pre- vs. post-synaptic sites, and as layer V neurons are the focus of most rodent mPFC recordings where there are few post-synaptic mGlu3 in rat, extra caution is needed when trying to translate mGlu3 data from rodent to primate species. However, there appear to be important post-synaptic mGlu3 actions in layer V of mouse mPFC that reduce connectivity with the amygdala and thalamus (Joffe et al., 2019a,b, 2020), as well as post-synaptic mGlu3 actions in the rodent dentate gyrus (Tamaru et al., 2001; Pöschel et al., 2005), indicating that non-traditional, post-synaptic mGlu3 may play especially interesting roles in cognitive circuits. Indeed, a recent study has shown that the activation of mGlu3 can enhance hippocampal function in mice (Dogra et al., 2021).

Finally, it should be noted that the macaque dlPFC and the rat mPFC differ not only by species, but also in PFC subregion. As the rodent does not have a dlPFC, the mPFC is often used as a “homolog,” given its role in working memory performance. However, the mPFC in rodents should ideally be compared to the mPFC in primates. The expression patterns of mGlu3 in macaque mPFC are currently not known, and would be another important area for future research.

### Roles of mGlu3 in Working Memory

Data from both macaque and the rodent suggest that mGlu3 signaling in PFC can benefit working memory. In monkey dlPFC, mGlu3 stimulation enhances mental representations of visual space held in working memory during the delay period by increasing delay-related firing for the neurons’ preferred direction (“signal”) vs. firing to the memory of non-preferred directions (“noise”) (Jin et al., 2018). The increased firing for the neurons’ preferred direction involved mGlu3 inhibition of detrimental cAMP-K^+^ actions, likely occurring on dendritic spines (Jin et al., 2018). It is possible that similar mechanisms occur in rat mPFC, as infusion of the GCPII inhibitor, 2-MPPA, directly into the rat mPFC, improved working memory performance (Datta et al., 2021). It is also possible that presynaptic/axonal and astroglial mGlu3 actions contribute to superior working memory function, where reduction of glutamate volume transmission may help to refine representations held in working memory by reducing “noise” (Jin et al., 2018). Future studies could examine whether mGlu3 receptor stimulation with NAAG enhances persistent firing in rat mPFC as it does in primates.

Studies using non-selective mGlu2/3 agents also support a role in working memory, where agonists increase the expression of phosphorylated NMDA glutamate receptors (NMDAR) in rat mPFC and ameliorate dysfunction caused by NMDAR blockade (Xi et al., 2011), while antagonist infusion impaired the working memory performance (Hernandez et al., 2018). Low doses of an mGlu2/3 agonist can also enhance Delay firing and working memory performance in monkeys, with local or systemic administration, respectively, consistent with low doses engaging post-synaptic mGlu3 and high doses engaging presynaptic mGlu2 actions (Jin et al., 2017). Low, but not high, doses of an mGlu2/3 agonist were found to be helpful in patients in the early stages of schizophrenia (Kinon et al., 2015), suggesting that strategies to enhance mGlu3 signaling may help treat cognitive disorders.

### Roles of mGlu3 in Glia

Metabotropic glutamate receptors type 3 expressed on glia also play key roles that may be especially important under conditions of injury and/or inflammation. For instance, studies of rodent cell cultures show that there is an increased expression of mGlu3 in microglia in response to inflammation, which prevents the microglia from becoming activated and neurotoxic, e.g., when exposed to myelin (Pinteaux-Jones et al., 2008), lipopolysaccharides (Berger et al., 2012), or IL-1ß (Zinni et al., 2021). Reactive astrocytes also upregulate their expression of mGlu3 (Berger et al., 2012), e.g., as seen in post-mortem tissue from patients with amyotrophic lateral sclerosis (Aronica et al., 2003). The roles of astrocytic and microglial mGlu3 remains an exciting avenue of research that requires further study to fully dissect how expression may be regulated in homeostasis and disease.

In summary, rat layer III mPFC shares some features of layer III dlPFC in primates, including glial expression; however, the predominant presynaptic labeling in rodent is strikingly different, where this role is performed by mGlu2 in the macaque. Thus, caution is needed when using the rodent mPFC as a model of mGlu3 dlPFC signaling in humans.

## Data Availability Statement

The raw data supporting the conclusions of this article will be made available by the authors, without undue reservation.

## Ethics Statement

The animal study was reviewed and approved by the Yale University IACUC.

## Author Contributions

EW and DD collected the data. All authors analyzed the data, wrote the manuscript, and contributed to this research.

## Conflict of Interest

Yale University and AA received royalties from Shire/Takeda from the USA sales of Intuniv (extended release guanfacine). They do not receive royalties from international sales, nor from sales of generic Intuniv. The remaining authors declare that the research was conducted in the absence of any commercial or financial relationships that could be construed as a potential conflict of interest.

## Publisher’s Note

All claims expressed in this article are solely those of the authors and do not necessarily represent those of their affiliated organizations, or those of the publisher, the editors and the reviewers. Any product that may be evaluated in this article, or claim that may be made by its manufacturer, is not guaranteed or endorsed by the publisher.
